# Estradiol metabolism by gut microbiota in women’s depression pathogenesis: inspiration from nature

**DOI:** 10.3389/fpsyt.2025.1505991

**Published:** 2025-01-28

**Authors:** Wei Zhang, Jinghan Jia, Yuhang Yang, Dawei Ye, Yan Li, Di Li, Jinxi Wang

**Affiliations:** ^1^ Division of Colorectal Surgery, Third Hospital of Shanxi Medical University, Shanxi Bethune Hospital, Shanxi Academy of Medical Sciences Tongji Shanxi Hospital, Taiyuan, China; ^2^ Tongji Hospital, Tongji Medical College, Huazhong University of Science and Technology, Wuhan, Hubei, China; ^3^ Neurology, Third Hospital of Shanxi Medical University, Shanxi Bethune Hospital, Shanxi Academy of Medical Sciences Tongji Shanxi Hospital, Taiyuan, China; ^4^ Department of Laboratory Medicine, Tongji Hospital, Tongji Medical College, Huazhong University of Science and Technology, Wuhan, China

**Keywords:** gut microbiota, women, estradiol, depression, degradation products, nature

## Abstract

The recurrence and treatment resistance of depression remain significant issues, primarily due to an inadequate understanding of its pathogenesis. Recent scientific evidence indicates that gut microbiota influence estradiol metabolism and are associated with the development of depression in nonpremenopausal women. Integrating existing studies on the regulation of estradiol metabolism by microorganisms in nature and the relevance of its degradation products to depression, recent scientific explorations have further elucidated the key mechanisms by which gut microbiota catabolize estradiol through specific metabolic pathways. These emerging scientific findings suggest that the unique metabolic effects of gut microbiota on estradiol may be one of the central drivers in the onset and course of depression in non-menopausal women.

## Graphical Abstract

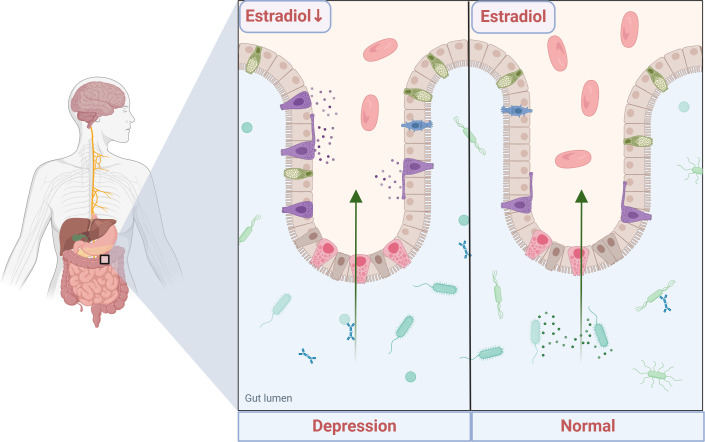

## 1 Introduction

Depression is a prevalent mental illness with potentially devastating consequences for individuals and society, characterized by prolonged depressed moods, negative thoughts, and fatigue (1). The World Health Organization (WHO) predicts that depression will become the most socioeconomically burdensome disease globally by 2030 (2). Depression is acknowledged to result from a combination of innate genetic predispositions and acquired environmental influences (3). In depressed patients, genetic factors and adverse childhood experiences are recognized as major contributors to depressed moods (4). Advancements in basic research have led to a preliminary understanding of the pathological mechanisms of depression, characterized by abnormalities in four main areas: brain function, the hypothalamic-pituitary-adrenal (HPA) axis, the immune system, and the gut-brain axis. Abnormalities in brain function primarily include neurotransmitter deficiencies (5), imbalances in Brain-Derived Neurotrophic Factor (BDNF) administration (6, 7), impaired neuroplasticity, and disruptions in neuronal circuits (8). Dysfunctions of the HPA axis are primarily characterized by dysregulation of the negative feedback mechanism (9, 10). Alterations in the immune system primarily involve increased expression of inflammatory molecules (11–13), while brain-gut dysfunction primarily relates to gastrointestinal disorders and abnormalities in the intestinal microbiota (14). Despite receiving appropriate treatment for depression, many patients remain vulnerable to relapse. Numerous studies (15–17) have demonstrated that, following initial successful treatment, 50% to 85% of individuals who have recovered from depression experience at least one recurrence of their condition. Moreover, additional research (18) indicates that patients may experience an average of approximately four relapses, and the risk of treatment resistance may increase with each recurrence of depression. This phenomenon could be attributed to the unclear understanding of the pathogenesis. There is a significant disparity in the prevalence of depression between the sexes, supported by statistical evidence indicating that women are approximately twice as likely as men to develop depression during their lifetime (19, 20). Research (21) has demonstrated that women experiencing depression during the premenopausal stage exhibit significantly lower levels of estradiol (E2) hormone compared to healthy women of the same age in control groups. Academic pioneers theorized over a century ago about a potential link between E2 and depression (22). The theory posits that maintaining appropriate levels of E2 in the blood is crucial for ensuring positive emotion regulation and mental health. Recent studies have reinforced the link between E2 and individual mood, particularly depression (23). E2 levels in women naturally decline during specific physiological transitions, such as postpartum recovery (24) and menopause (25), rendering them more susceptible to depressive symptoms during these periods. Around 3%-4% of women undergo declines in E2 levels unrelated to menopause, lactation, or pregnancy (26, 27), with primary ovarian insufficiency being the primary cause (28). Decreased E2 levels may result from various endocrine disorders, including congenital adrenal hyperplasia, hyperprolactinemia, and polycystic ovary syndrome. However, the precise mechanism and underlying cause of estrogen reduction in these conditions remain incompletely understood. Decreased E2 levels are often accompanied by abnormalities in the compositional abundance of intestinal microbiota (29). Depressed patients exhibit notable alterations in the composition of the intestinal microbiota, characterized by a relative increase in bacteria belonging to the phylum Aspergillus and Actinobacteria (30). Certain bacterial species within both phyla have been observed to degrade steroids in natural environments (31), implying their potential indirect involvement in the onset and progression of depression through interference with metabolic pathways of steroid radicals like E2 and the production of related metabolites.

Gut Microbiota (GM) encompasses the vast array of microorganisms inhabiting the human digestive tract, predominantly the colon. Comprising bacteria, fungi, viruses, and various other types, these microorganisms are abundant and diverse, collectively weighing between 1-2 kg. Widely acknowledged as a vital “organ” of the human body, GM plays an indispensable role in maintaining overall human health. The GM and the human body have co-evolved into a complex system of reciprocal symbiosis, where GM thrives in a mutually beneficial balance while contributing to the health of the human body (32–34). However, disruptions to this balance can lead to significant alterations in GM composition and abundance, resulting in microbial ecological imbalance. This disrupted state may be closely linked to an elevated risk of various health problems (35–38). Our recent studies have unveiled that gut microbes regulate estradiol activity, impacting serum E2 levels via 3β-hydroxysteroid dehydrogenase (3β-HSD) expression (39), closely linked to depression development. Notably, estradiol-degrading bacteria are present in nature, displaying intricate metabolic pathways for E2. These microorganisms synthesize various enzymes, converting E2 into diverse metabolites, potentially influencing depression onset and progression. These diverse metabolites may potentially influence the onset and development of depression. Therefore, a multidimensional and integrative analytical approach is necessary to investigate how the gut microbiota influences depression, particularly through the estradiol metabolic pathway. This paper examines four aspects: molecular mechanisms of E2 in depression, the role of microorganisms in E2 regulation, impact of estradiol degradation products in nature on depression, the E2-gut microbiota relationship, and the gut microbiota’s impact on depression through E2 metabolism.

## 2 The molecular mechanisms of estradiol in depression

**Figure 1:**
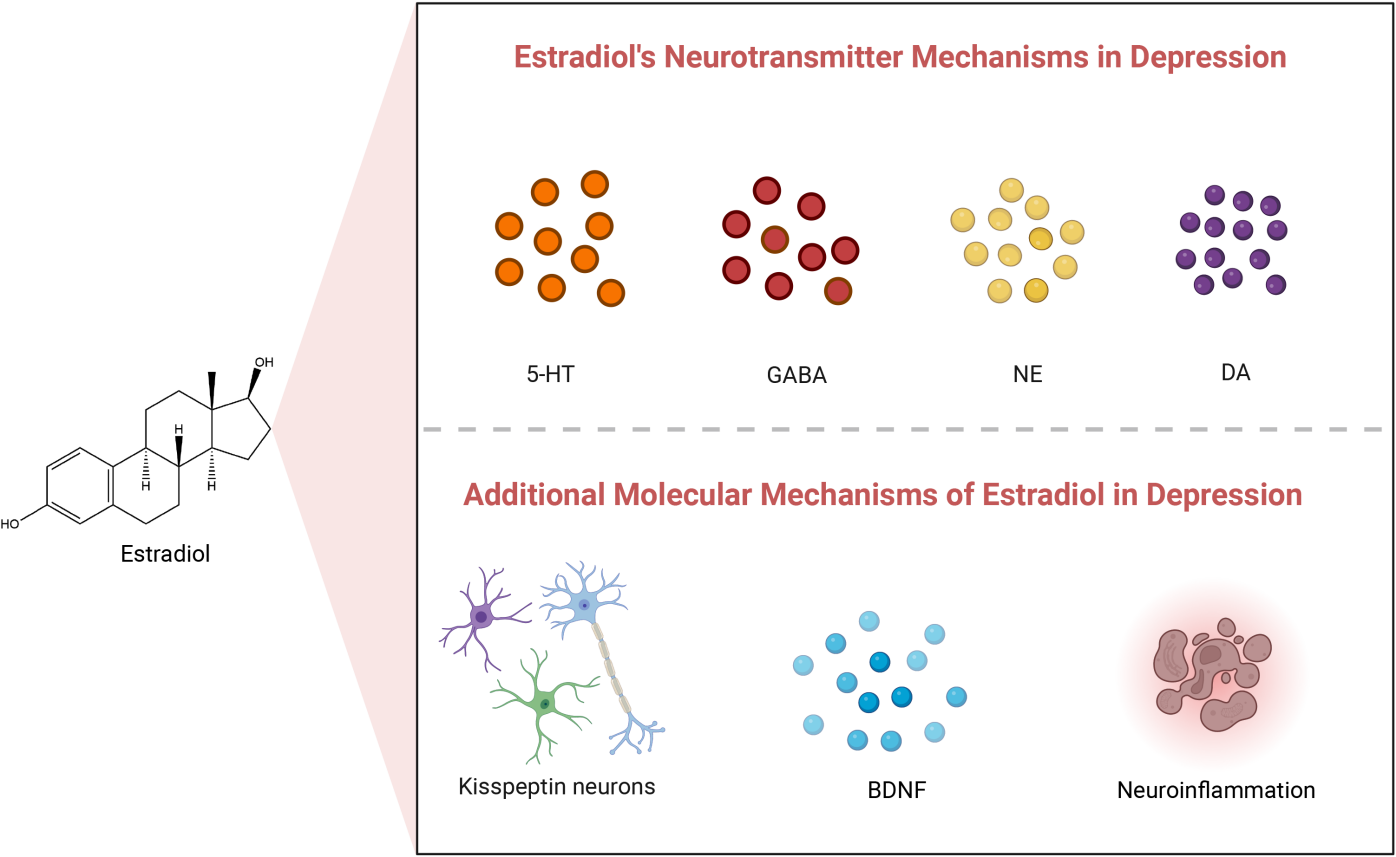
Estradiol’s neurotransmitter and other molecular mechanisms in depression. 5-HT, 5-Hydroxytryptamine; GABA, γ-Aminobutyric acid; NE, Norepinephrine; DA, Dopamine; BDNF, Brain-Derived Neurotrophic Factor.

E2 plays a pivotal endocrine regulatory role in premenopausal women, primarily synthesized from cholesterol in the ovaries, corpus luteum, and placenta. Ovarian sheath cells produce androgens but lack the capacity for estrogen production. Conversely, granulosa cells, housing aromatase, convert androgens from sphingocytes into E2. Sphingocytes and granulosa cells collaborate in various ways to synthesize and regulate ovarian estrogens. E2 is a vital steroid hormone widely recognized as the most biologically active estrogen. E2 is crucial for maintaining overall physiological function in women, with a broad spectrum of roles. It regulates the menstrual cycle’s regularity, supports normal development and function of reproductive organs, influences breast tissue development, and is vital for maintaining bone strength and cardiovascular health. Moreover, E2 acts as both a gonadal and neuroactive steroid hormone (40, 41). One of its primary functions is to influence mood regulation, cognitive function, and various other brain-related physiological processes by modulating the release of key neurotransmitters in the central nervous system, including 5-hydroxytryptamine (5-HT), γ-aminobutyric acid (GABA), norepinephrine (NE), and dopamine (DA) (42), as well as at other molecular levels (43, 44). **Table 1** provides a comprehensive summary of the molecular mechanisms underlying the impact of changes in estradiol concentration on the pathogenesis of depression. **Figure 1** illustrates the potential mechanisms of estradiol involvement in depression.

**Table 1:** Mechanisms of estradiol concentration changes and metabolites in the pathogenesis of depression.

Mechanism	Substance	Impact Mechanisms or Research Findings on Depression	References
Neurotransmitter Regulation	5-HT	- A decrease in E2 reduces 5-HT transmission efficiency and the expression of related genes, increasing the risk of depression. - An increase in E2 enhances postsynaptic 5-HT transmission by regulating 5-HT1A and 5-HT2A receptors, thereby reducing depression risk.	(46, 52, 55, 58)
	GABA	- E2 fluctuations significantly affect the expression and function of GABA receptors, disrupting emotional regulation.	(68, 69)
	NE	- A decrease in E2 reduces NE synthesis and increases its metabolic degradation, leading to emotional instability. - An increase in E2 significantly enhances NE metabolic turnover, improving cognitive and emotional regulation.	(71–73, 75)
	DA	- A decrease in E2 impairs dopamine synthesis and receptor activity, reducing emotional regulation capabilities. - E2 therapy restores D1 and D2 receptor density, stabilizing mood and cognitive function.	(77, 78, 80)
Neuroplasticity and Nutrition	Kisspeptin Neurons	- A decrease in E2 suppresses Kiss1 gene expression, increasing the risk of emotional instability. - An increase in E2 significantly enhances Kiss1 expression, reducing the risk of depression.	(88, 89, 91)
	BDNF	- A decrease in E2 reduces BDNF transcription and translation, significantly increasing the risk of perinatal and postpartum depression. - BDNF levels positively correlate with E2, peaking during ovulation and potentially restored through hormone replacement therapy (HRT).	(96, 98–102)
Inflammation and Neuroprotection	Neuroinflammation	- E2 suppresses local inflammation, promotes glial cell production, and regulates the release of neurotrophic factors, improving depressive-like behaviors. - E2 synergizes with insulin-like growth factor 1 (IGF-1) to activate anti-inflammatory and neuroprotective pathways, enhancing its antidepressant effects.	(107, 108, 113–115)
Estradiol Metabolites	E1	- E1, a key component of hormone replacement therapy (HRT), alleviates perimenopausal symptoms but exhibits weaker effects on neuronal survival. - The highly active form of E2 shows greater protective effects against depression compared to E1.	(166, 167, 170)
	DHT	- DHT improves cognitive and motor functions in neuroinflammatory models, although its levels may correlate with the severity of depressive symptoms in certain individuals.	(172, 173)
	4-OH-E1	- Although its estrogenic activity is relatively weak, 4-OH-E1 exhibits significant protective effects against oxidative stress-induced neurotoxicity, even surpassing highly active E2 in some cases.	(174)
	4-OH-E2	- Increases NE levels, demonstrating significant antidepressant effects.	(178)

5-HT, 5-Hydroxytryptamine; GABA, γ-Aminobutyric acid; NE, Norepinephrine; DA, Dopamine; BDNF, Brain-Derived Neurotrophic Factor; E1, Estrone; DHT, Dihydrotestosterone; 4-OH-E1, 4-hydroxyestrone; 4-OH-E2, 4-hydroxyestradiol.

### 2.1 The impact of estradiol on neurotransmitters

#### 2.1.1 5-Hydroxytryptamine

5-HT, commonly known as serotonin, plays diverse roles in the central and peripheral nervous systems. It was first isolated and identified by Maurice Rapport and Irvine Page in 1948 (45). In the central nervous system, 5-HT interacts extensively with the 5-hydroxytryptaminergic nervous system, which spans the forebrain, brainstem, and cerebellum. Neural projections from the anastomosing lateral nucleus regulate fundamental physiological functions, including temperature regulation, appetite control, sleep-wake cycles, vomiting, and sexual behavior. Meanwhile, projections to the caudate nucleus are linked to pain perception and motor tone regulation (46). Additionally, 5-HT is essential for emotional stability (47), and disruptions in 5-HT function can lead to depression, impulsive behaviors, and, in some cases, a heightened risk of suicide (48).

Pathophysiological studies have further explored serotonin’s role in emotional regulation, with reduced transmission efficiency identified as a key hypothesis for depression mechanisms (49). Research supports this, showing that estrogen (E2) regulates gene expression by binding to the endoplasmic reticulum, a process validated in cell line models (50). This regulation is particularly evident in brain regions such as the nucleus accumbens, where 5-HT neurons are abundant (51). Furthermore, studies reveal that E2 enhances postsynaptic 5-HT transmission by downregulating 5-HT1A autoreceptors and upregulating 5-HT2A receptors (52). These findings form the foundation for understanding how E2 modulates 5-HT neurotransmission.

In mammals, 5-HT is widely distributed, with approximately 90% synthesized and concentrated in intestinal tissues (53, 54). Animal models have been instrumental in examining the relationship between serotonin, depression, and emotional regulation. For example, estrogen deficiency induced by ovariectomy in monkeys led to decreased 5-HT efficacy and reduced expression of 5-HT-related genes compared to controls (55). Moreover, research shows that E2 selectively increases serotonin receptor density in brain regions containing estrogen receptors, including the hypothalamus, preoptic area, and amygdala (56). These findings suggest that E2-mediated regulation of 5-HT transmission may play a crucial role in protecting against depression.

Clinical studies further highlight the association between 5-HT concentration and psychiatric disorders such as depression, mania, and anxiety. Kandel et al. demonstrated that reduced 5-HT concentration in the central nervous system significantly correlates with the onset of these conditions (57). Specifically, patients with depression and suicidal tendencies exhibit lower 5-HT1A mRNA expression in the hippocampus compared to non-depressed individuals (58). These observations underscore serotonin’s vital role in emotional and behavioral regulation and provide a basis for its therapeutic application in depression treatment.

Early antidepressant therapies, including Monoamine Oxidase Inhibitors (MAOIs) and Tricyclic Antidepressants (TCAs), improved neurotransmitter availability in synaptic gaps by targeting 5-HT, dopamine (via DAT), and norepinephrine (via NET). Subsequent research has refined these approaches, with serotonin-specific drugs demonstrating greater efficacy. However, scholar Sameer Jauhar (59) has argued that while these drugs modulate serotonin effectively, they often fail to address underlying etiological factors of depression.

Research across various experimental platforms emphasizes that serotonin’s role extends beyond emotional regulation, as it is influenced by numerous factors. Estrogen’s modulation of gene expression and its regulation of 5-HT receptors, for example, likely contribute to its protective effects against depression. Collectively, these studies indicate that serotonin system dysfunction is linked to multiple psychiatric disorders and provide an experimental basis for developing future therapeutic strategies.

#### 2.1.2 γ-Aminobutyric acid

GABA (γ-aminobutyric acid) is the primary inhibitory neurotransmitter widely distributed throughout the mammalian central nervous system (CNS) (60). Approximately 30% of central nervous fibers utilize GABA as a neurotransmitter. Although GABAergic neurons constitute only a small proportion of the total neuronal population, their inhibitory functions and balance with excitatory transmission are critical for maintaining normal brain activity. GABA receptors are highly distributed throughout the nervous system, particularly on postsynaptic membranes, as well as in the cerebellum and hippocampus. GABA primarily exerts its physiological functions through three receptor types: the ion channel receptors GABAA and GABAC, and the metabotropic receptor GABAB (61).

In recent years, research has highlighted the critical role of GABAergic system dysfunction in the neurobiological mechanisms of depression (62–64). Fundamental studies have demonstrated that as a metabotropic receptor, the GABAB receptor plays a pivotal role in regulating postsynaptic signal transduction, and its dysfunction is regarded as an important target in the pathological processes of depression (61, 65). Additionally, Kelly et al.’s cell line studies suggest that estrogen (E2) may exert antagonistic effects on GABAA receptors, indicating that E2 might regulate emotional states and brain functions associated with depression through GABA signaling (66).

Animal model studies have provided significant experimental evidence of E2’s regulatory effects on the GABA system (67). E2 inhibits presynaptic GABA release via a rapid nongenomic mechanism, a phenomenon prominently observed in female mice but not in male mice. This sex-specific difference may be closely associated with variations in the production of inositol 1,4,5-triphosphate (IP3) and the activation state of its corresponding receptor (IP3R) (68). The regulatory effects of E2 significantly influence GABAergic neurotransmission in key brain regions, such as the prefrontal cortex and hippocampus, offering potential explanations for the molecular mechanisms underlying the sex differences in depression.

Clinical studies further indicate that during the critical periods of steroid hormone-mediated brain sexual differentiation, the GABA system is highly sensitive to E2 (69). For instance, during puberty and perimenopause, fluctuations in E2 levels significantly impact the expression and function of GABA receptors, particularly in brain regions associated with emotional regulation. These findings suggest that E2 may play a vital role in the pathological processes of depression by modulating GABAergic transmission and synaptic plasticity.

#### 2.1.3 Norepinephrine

Norepinephrine (NE), a critical neurotransmitter and hormone, plays a vital role in the central nervous system through various molecular mechanisms. Experimental studies demonstrate that NE functions primarily via the α- and β-adrenergic receptor families, which regulate essential neural activities, including alertness, arousal, attention, and motivation-driven behaviors (70). Research also indicates that estrogen modulates NE activity by influencing the distribution and expression of these receptors (71). Additionally, 17β-estradiol (E2) indirectly increases NE levels by inhibiting monoamine oxidase expression, thereby reducing NE’s metabolic degradation (72). E2 further enhances tyrosine hydroxylase activity, a key enzyme in the catecholamine synthesis pathway, promoting NE synthesis (73). These findings provide critical insights into NE’s regulatory mechanisms at both the molecular and receptor levels.

Animal studies offer additional evidence of how estrogen regulates NE neurotransmission and its impact on emotional and cognitive functions. In ovariectomized mouse models, estrogen receptors are expressed in norepinephrine neurons, which project to multiple brain regions, including the hypothalamus, a critical area for emotional regulation (72, 74). E2 treatment significantly increases the metabolic turnover rate of NE in the hypothalamus and cerebral cortex (75). These results suggest that estrogen influences NE levels through multiple mechanisms, profoundly affecting emotional regulation and cognitive processes.

Clinical studies further support the interplay between NE and estrogen in emotional regulation. Research shows that fluctuations in estrogen levels are closely associated with the onset and progression of depressive symptoms (72, 74). During perimenopausal and postpartum periods, declining estrogen levels may reduce NE utilization efficiency, contributing to emotional instability. Clinical evidence also reveals that E2 enhances NE synthesis and utilization while reducing its metabolic degradation, alleviating depressive symptoms in patients. Additionally, estrogen modulates adrenergic receptor function, significantly influencing reward mechanisms and cognitive functions, including attention (71). These findings provide a foundation for developing targeted interventions to regulate NE in depressive disorders.

#### 2.1.4 Dopamine

Dopamine (DA), a key neurotransmitter in the central nervous system (CNS), belongs to the catecholamine family and is closely linked to the pathophysiology of depression (76). Synthesized and released by dopaminergic neurons, DA acts through five major receptor subtypes: D1, D2, D3, D4, and D5. Among these, D1, D2, and D3 receptors are regulated by E2 (17β-estradiol). These receptors are unevenly distributed across brain regions and play critical roles in motor control, emotional processing, cognitive function, motivation, reward mechanisms, and endocrine regulation.

Basic research has demonstrated that E2 regulates the DA system by upregulating the mRNA expression of the long isoform of the D2 receptor, thereby enhancing DA neurotransmission. This effect is primarily observed in the midbrain, where DA neurons are highly concentrated, indicating that E2 directly modulates DA synthesis and utilization at the molecular level (77).

Animal studies further emphasize E2’s extensive regulatory effects on the DA system and its role in physiological processes. In ovariectomized animal models, the density of D1 and D2 receptors in the striatum is significantly reduced, while D3 receptor density remains unchanged (77–79). E2 treatment restores D1 and D2 receptor density, indicating its ability to modulate postsynaptic receptor function and normalize DA system activity (78). Furthermore, E2’s effects are not confined to specific regions. One study shows that E2 affects not only the hypothalamus but also extra-hypothalamic brain areas involved in motor function and behavior regulation, highlighting its broad regulatory role in the DA system (78).

Clinical studies show that DA activity declines with age, potentially contributing to the onset of depression (80). Additionally, multiple studies report that DA D1 and D2 receptor activity in the striatum fluctuates cyclically during the menstrual cycle (81–83). These fluctuations closely correspond to changes in estrogen levels, further supporting E2’s regulatory influence on DA receptors. Furthermore, E2 enhances DA utilization and significantly affects emotional regulation. These findings provide critical evidence for the potential application of E2 in treating mood disorders such as depression.

### 2.2 Additional molecular mechanisms of estradiol in depression

#### 2.2.1 Kisspeptin neurons

Kisspeptin (Kiss1) neurons play a central role in the sex steroid feedback mechanism within the human hypothalamus. Current research on Kisspeptin neurons predominantly focuses on animal models and clinical studies, with limited investigation at the basic molecular level. Studies of Kisspeptin receptor signaling pathways suggest that estrogen regulates the activity of Kisspeptin neurons through molecular mechanisms, shedding light on their integration within neural networks.

Animal studies have demonstrated that Kisspeptin and its receptors are essential for regulating sex hormones and related behaviors. Kiss1 neurons are primarily located in the lateral septum, the bed nucleus of the stria terminalis (BNST), and the medial amygdala (84). These neurons stimulate the release of gonadotropins by responding to sex steroid signals, increasing the pulsatile activity of gonadotropin-releasing hormone (GnRH) and luteinizing hormone (LH), a mechanism observed in both sexes (85, 86). Furthermore, Kisspeptin signaling is associated with behaviors such as anxiety and depression (87). Estradiol (E2) significantly increases Kiss1 expression in the preoptic area (POA) of both male and female monkeys (88, 89). In ovariectomized young monkeys, researchers observed hypertrophy in basal ganglia neurons of the hypothalamus, a phenomenon also identified in postmenopausal women (90). Conversely, E2 deficiency markedly suppresses Kiss1 expression, underscoring the detrimental effects of reduced estrogen levels on Kisspeptin neurons (91).

Clinical studies further emphasize the critical role of Kisspeptin neurons in the sex steroid feedback mechanism. A specific population of Kiss1 neurons in the lateral hypothalamus decreases after menopause, suggesting that estrogen positively regulates neuronal activity and homeostasis in this brain region (92). Additionally, basal nucleus neurons in the hypothalamus of postmenopausal women exhibit enlargement alongside a significant increase in Kisspeptin gene expression (85, 93). These findings highlight the regulatory influence of estrogen on Kiss1 neurons, their role in modulating GnRH secretion, maintaining reproductive function, and promoting emotional stability.

In conclusion, Kiss1 neurons are key regulators of GnRH secretion and demonstrate significant sensitivity to fluctuations in estrogen levels, as shown in animal and clinical studies. These findings underscore their central role in the sex steroid feedback mechanism. However, further studies using cellular models and primary culture systems are necessary to elucidate the molecular regulatory mechanisms of Kisspeptin neurons, advancing our understanding of their roles in neural and hormonal regulation.

#### 2.2.2 Brain-derived neurotrophic factor

BDNF is a protein widely expressed in the brain and classified within the neurotrophin family. It is secreted by various cell types in the central nervous system (CNS), including neurons, astrocytes, and microglia (94). BDNF plays a critical role in physiological processes such as cognition, memory, and emotional regulation (94). Additionally, it is essential for neuronal growth, differentiation, survival, and synaptic plasticity.

Research using cell lines and primary cultures has elucidated the molecular mechanisms of BDNF production. For instance, *in vitro* studies reveal that activation of the MAPK signaling pathway enhances BDNF gene transcription via CREB phosphorylation, further supporting the regulatory influence of estrogen on BDNF expression (95). These findings provide direct evidence of the molecular and signaling pathways involved in BDNF regulation.

Animal model studies have shown that changes in BDNF levels are linked to specific physiological and pathological states. For example, research on ovariectomized mice demonstrates a significant reduction in BDNF transcription and translation in the brain, suggesting that BDNF is regulated by sex hormones (96). Moreover, the BDNF gene contains a homologous sequence of estrogen response elements (ERE), which mediates estrogen’s effects on BDNF gene expression (97).

Clinical studies further confirm the connection between BDNF levels and depression. Evidence shows that reduced BDNF levels significantly increase the risk of depression during pregnancy and the postpartum period (98–100). In non-pregnant women, serum BDNF levels correlate positively with estradiol (E2) levels, with both peaking during ovulation (101). Additionally, postmenopausal women experience decreased BDNF levels, which can be restored through hormone replacement therapy (102).

In conclusion, research across multiple models—including animal studies, clinical investigations, cell line experiments, and primary cultures—consistently underscores the vital role of BDNF in cognitive regulation, depression, and hormone-mediated modulation. These findings, based on multi-level experimental approaches, offer valuable insights into the complex functions and regulatory mechanisms of BDNF in the nervous system.

#### 2.2.3 Neuroinflammation

Acute neuroinflammation initially functions as a temporary physiological mechanism to defend and repair neural tissue damage. However, in chronic neurodegenerative diseases and aging, neuroinflammation transitions into a persistent pathological state. This prolonged inflammatory response not only fails to protect neural tissue but also exacerbates neuronal damage and disrupts the overall neural structure (103–105).

Animal studies provide robust evidence linking neuroinflammation to neurodegenerative diseases and depressive-like behaviors. For example, experiments in animal models have demonstrated that central nervous system (CNS) inflammation can induce depressive-like behaviors (106). Additionally, chronic neuroinflammation has been shown to impair neuronal survival and function, highlighting its central role in these pathological conditions.

Basic research has clarified the mechanisms through which estradiol (E2) modulates neuroinflammation and apoptosis. Studies using primary cultures of astrocytes and microglia reveal that E2 suppresses local inflammation, promotes gliogenesis, and regulates the release of neurotrophic factors to improve the neural environment (107, 108). Moreover, cell-based studies indicate that E2 enhances the expression of molecules critical for neuronal survival via estrogen receptor subtypes, including ERα, ERβ, and G-protein-coupled receptors (GPCRs), while concurrently downregulating pro-apoptotic and pro-inflammatory molecules (109–111).

Clinical research further supports the neuroprotective and antidepressant properties of E2. Evidence suggests that E2 preserves neurological health by mitigating inflammation and inhibiting apoptosis (112). Furthermore, clinical studies demonstrate that E2 synergizes with insulin-like growth factor 1 (IGF-1) to activate neuroprotective and anti-inflammatory signaling pathways, thereby amplifying its neuroprotective and antidepressant effects (113–115).

In conclusion, E2 may significantly contribute to depression in premenopausal women through various known pathways and potentially unknown mechanisms. Thus, appropriate monitoring and regulation of E2 levels are crucial when addressing and managing the mental health of non-menopausal women.

## 3 Microbial degradation of estradiol in natural environments

### 3.1 Microbial degradation of estradiol in natural environments

Researchers have identified and characterized numerous microbial strains capable of degrading E2 in various natural environments, including activated sludge (116), compost (117), soil sediments (118), and sandy groundwater layers (119). The following strains and fungi are known for their ability to degrade E2: *Denitratisoma* sp. *strain DHT3*, *Denitratisoma oestradiolicum AcBE2-1^T^*, *Microbacterium strain KC8*, *Steroidobacter denitrificans FS^T^*, *Aminobacter strains KC6 and KC7*, *Ralstonia pickettii*, *Sphingomonas* sp. *ED8*, *Novosphingobium* sp. *ARI-1*, *Rhodococcus* spp. *strain KC4*, and White rot fungi Trametes versicolor. Additionally, microbial degradation products of E2 include, but are not limited to, estrone (E1), dihydrotestosterone (DHT), 4-hydroxyestrone(4-OH-E1), 4-hydroxyestradiol(4-OH-E2), and others.

**Figure 2:**
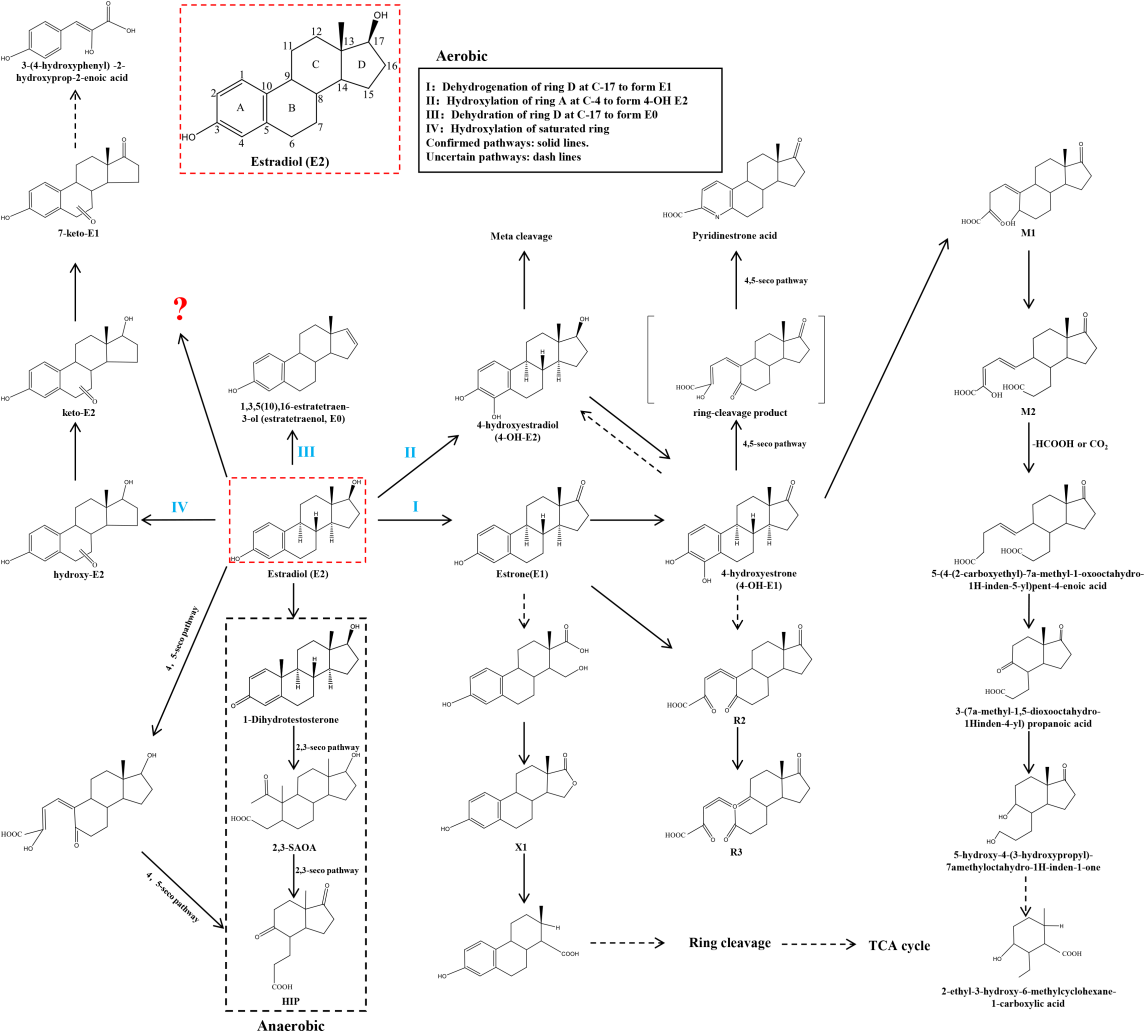
Microbial pathways for E2 degradation in nature. The aerobic pathway is divided into 4 types. I: Dehydrogenation of ring D at C-17 to form E1. II: Hydroxylation of ring A at C-4 to form 4-OH E2. III: Dehydration of ring D at C-17 to form E0. IV: Hydroxylation of saturated ring. The anaerobic pathway is the generation of 1-Dihydrotestosterone.? for the unknown pathway of E2 degradation. Confirmed pathways: solid lines. Uncertain pathways: dash lines.

The biological effects of E2 rely on the integrity of its molecular structure. Any chemical transformations or metabolic changes undergone by E2 typically result in alterations to its biological activity. Since the specific mechanism of E2 degradation by gut microorganisms is not yet fully understood, information on naturally occurring microorganisms capable of degrading E2 will be gathered from the published literature. This includes details on the reaction steps involved, the metabolites produced, and the key enzymes catalyzing the process. These enzymes are classified as dehydrogenases (e.g., cobalamin-dependent methyltransferase, 17β-hydroxysteroid dehydrogenase), monooxygenases (e.g., estrone 4-hydroxylase, nonspecific monooxygenase, ammonia monooxygenase), dioxygenases (e.g., 4-hydroxyestrone 4,5-dioxygenase), and other oxidases (e.g., laccase, cytochrome oxidase). Such data will provide an important theoretical basis and guide research efforts toward understanding how gut microorganisms degrade E2. In **Supplementary Table 1**, we will systematically list several strains capable of degrading E2, along with their associated metabolites and enzymes. To delve deeper into the degradation mechanisms of these microorganisms, we will classify them into three categories: aerobic pathways, anaerobic pathways, and unknown pathways, based on documented degradation pathways in the literature. This classification will provide a comprehensive analysis and discussion of the microorganisms. Refer to **Figure 2** for visual representation.

#### 3.1.1 Aerobic pathway

Forty years ago, Coombe et al. (120) documented the initial degradation pathway of E1 by the soil bacterium *Nocardia* sp. *E110*. Their study identified a dioxygenase that crucially catalyzes the cleavage of the ‘a’ region within the ring structure of the E1 molecule. This pathway has been integrated into the aerobic E2 degradation pathway, which has been uncovered in recent years. The initial stages of E2 degradation entail four distinct processes: (I) dehydrogenation of the D-ring at C-17; (II) hydroxylation of the A-ring at C-4; (III) dehydration of the D-ring at C-17; and (IV) hydroxylation of the saturated ring.

##### 3.1.1.1 Dehydrogenation of the D-ring at C-17

Research has demonstrated that E2 can undergo conversion to E1 during biodegradation (121). This conversion entails dehydrogenation at the C-17 position of the D-ring in the E2 molecule, resulting in the formation of E1. Furthermore, E1 can undergo hydroxylation at the C-4 position of the A-ring, leading to the formation of its metabolite, 4-OH-E1, which is susceptible to subsequent biodegradation through the metabolic cleavage pathway (118).

The following microorganisms, gathered from the literature, are capable of converting E2 to E1.

*Sphingomonas strain KC8 (* 122–124) rapidly degrades E2 and E1 into estrogenic metabolites. E1 is the primary product of estradiol degradation catalyzed by 17β-hydroxysteroid dehydrogenase. Subsequently, flavin-dependent monooxygenase (KCB_16650), encoded in cluster I of the *Sphingomonas strain KC8* gene, degrades E1. E1 is initially produced in estradiol degradation. Subsequently, it undergoes conversion to 4-hydroxyestrone by flavin-dependent monooxygenase (estrone4-hydroxylase), encoded by oecB (KCB_16650) in cluster I of the KC8 gene of *Sphingomonas strain KC8*. 4-hydroxyestrone undergoes ring cleavage catalyzed by extradiol dioxygenase (4-hydroxyestrone4,5-dioxygenase), encoded by cluster oecC (KC8_05325) in the Sphingomonas strain KC8 gene II. Subsequently, this compound undergoes nonenzymatic transformation to produce pyridinestrone acid, a characteristic marker of the 4,5-seco pathway. *Acinetobacter* sp. *DSSKY-A-001 (* 125) is capable of degrading E2 to E1, and subsequently to R2 and R3. Literature suggests that 4-OH-E2 and 4-OH-E1 are mutually interconvertible. Additionally, E1 can be further transformed into 4-OH-E1, and then converted to R2 with the addition of one oxygen molecule. Although 4-OH-E1 and 4-OH-E2 were not detected in the literature, it is plausible that E1 can directly convert to R2 by incorporating two oxygen molecules. The enzymes involved in these processes may include catechol 1,2-dioxygenase, dioxygenase, and 7-α-hydroxysteroid dehydrogenase. *Rhodococcus* sp. *DS201 (* 126) has been documented in the literature as capable of degrading E2, with various E2 metabolites identified through high-performance liquid chromatography (HPLC). These metabolites include E1, 4-OH-E1, M1, M2, 5-(4-(2-carboxyethyl)-7a-methyl-1-oxooctahydro-1H-inden-5-yl)pent-4-enoic acid, pent-4-enoic acid, 3-(7a-methyl-1,5-dioxooctahydro-1H-inden-4-yl)propanoic acid, 5-hydroxy-4-(3-hydroxypropyl)-7a-methyloctahydro-1H-inden-1-one, and 2-ethyl-3-hydroxy-6-methylcyclohexane-1-carboxylic acid. Additionally, a novel metabolic pathway has been proposed for one of these metabolites. *Sphingomonas* sp. *ED8 (* 118) oxidizes E2 to E1 via an oxidase enzyme, followed by the conversion of E1 to 4-OH-E1. *Aminobacter strains KC6* and *KC7 (* 124), belonging to the Aminobacteria class, are estradiol-degrading bacteria isolated from activated sludge in wastewater treatment plants. Both exhibit non-specific monooxygenase activity and can degrade both E2 and E1. However, the literature (124) only documents E1 as one of the products generated from the degradation of E2 by these strains, leaving unclear the subsequent products of E1 degradation. *Sphingomonas* sp. *CYH (* 119), *Ralstonia pickettii BP2 (* 117), and *Phyllobacterium (* 117) have also been reported to possess a transformation process that converts E2 to E1. The E1 produced in this process is capable of further degradation. However, the literature lacks exhaustive information regarding the specific products resulting from the subsequent degradation of E1. The following strains have been identified for their ability to degrade E2: *Flavobacterium strain KC1, Nocardioides strain KC3, Rhodococcus strain KC4, Microbacterium strain KC5, Sphingomonas strains KC9, KC10, KC11, KC14, Brevundimonas strain KC12, Escherichia strain KC13 (* 124), *Brevundimonas diminuta I (* 127), *Virgibacillus halotolerans LF1, Bacillus flexus LF3, Bacillus licheniformis LF5 (* 128), and *Bacillus* sp. *E2Y1, E2Y2, E2Y3, E2Y4, E2Y5 (* 129). However, the degradation products of E2 in all cases consist of E1 and do not undergo further degradation. An alternative degradation pathway for E2 was uncovered in a study by Lee et al. (130). This pathway revealed a new metabolite, X1, identified for its lactone structure on the D-ring, discovered while investigating bacteria in mixed effluents. The hypothesis proposed that metabolite X1 could undergo further degradation by integration into the tricarboxylic acid cycle (TCA cycle).

##### 3.1.1.2 Hydroxylation of the A-ring at C-4

Kurisu et al. (118) proposed a pathway for the conversion of E2 to E1 by *Sphingomonas* sp. *ED8*. However, this represents just one degradation pathway for E2 by this bacterium. Additionally, they identified another degradation pathway for E2 by *Sphingomonas* sp. *ED8*. Furthermore, they discovered the intermediate metabolite 4-OH-E2, demonstrating hydroxylation of E2 at the C-4 position of the A-ring. They suggested that 4-OH-E2 could undergo further degradation via meta-cleavage.

##### 3.1.1.3 Dehydration of the D-ring at C-17

Shi, Nakai, and colleagues (131) reported in a 2004 study that the *ammonia-oxidizing bacterium Nitrosomonas europaea* significantly degraded E1, E2, E3, and Ethynyl Estradiol (EE2). They found that the rate of E2 degradation was notably diminished in the presence of ammonia-oxidizing inhibitors. This suggests that ammonia monooxygenase (AMO) likely plays a role in estrogen degradation. However, despite their efforts, the investigators were unable to identify the specific metabolites of these strains after the degradation of E2. It was evident, though, that they were unable to directly convert E2 to E1. Until 2011 (132), Nakai and Shi discovered a new intermediate metabolite, 1,3,5 (10),16-tetraen-3-ol (estratetraenol or E0), formed by the dehydration of E2 at its D-ring C-17. Although E0 retains some estrogenic activity, subsequent research demonstrated that the ammonia-oxidizing bacterium Nitrosomonas europaea can further degrade E0 into compounds devoid of estrogenic activity.

##### 3.1.1.4 Hydroxylation of the saturated ring

Kurisu and colleagues (118) not only observed the conversion of E2 to E1 and 4-OH-E2 in the degradation pathway of *Sphingomonas* sp. *ED8* but also identified hydroxy-E2, keto-E2, 7-keto-E1, and 3-(4-hydroxyphenyl)-2-hydroxyprop-2-enoic acid. This confirms that E2 can undergo degradation through hydroxylation reactions at various ring positions. Remarkably, the detection of 3-(4-hydroxyphenyl)-2-hydroxyprop-2-enoic acid indicates that the degradation of E2 initiates from the B, C, or D rings of the saturated ring, rather than the A ring. However, scholars did not provide insights into the activation and occurrence of this crucial step. The chemical mechanism of how this key step, involving the saturated ring, is activated and cleaved, remains largely unexplored. Consequently, further scientific research is necessary to elucidate the detailed molecular mechanism of this pivotal aspect of the degradation pathway.

#### 3.1.2 Anaerobic pathway

*Denitratisoma* sp. *strain DHT3*, also referred to as the denitrosomal strain DHT3 (133), is a denitrifying bacterium known for its capability to degrade E2. Under anaerobic conditions, it enzymatically converts E2 to the primary product DHT through cobalamin-dependent methyltransferase-mediated methylation. Subsequently, it further metabolizes the androgenic intermediate via the established 2,3-seco pathway, yielding 17β-hydroxy-1-oxo-2,3-seco-androstan-3-oic acid (2,3-SAOA) and 3aα-H-4α(3′-propanoate)-7aβ-methylhexahydro-1,5-indanedione (HIP). Additionally, the text mentions the 4,5-seco pathway of estradiol degradation under anaerobic conditions, where the end products are funneled to HIP.

#### 3.1.3 Unknown degradation pathways

*Denitratisoma oestradiolicum AcBE2-1^T^ (* 134) is a Gram-negative denitrifying bacterium. It was isolated from activated sludge of municipal wastewater treatment plants and utilizes 17β-estradiol as its sole carbon and energy source. This bacterium exhibits the ability to thoroughly and completely biotransform E2 through the action of cytochrome oxidase. Consequently, E2 compounds undergo complete degradation and are ultimately converted into CO_2_ and H_2_O during this anaerobic oxidation process. White rot fungi, a group of fungi capable of effectively degrading lignin and various environmental pollutants, were first reported by Suzuki et al. (135) to degrade E2 efficiently through ligninolytic enzymes and laccase, consequently removing its estrogenic activity. However, the researchers observed that no metabolites were detectable in the reaction mixture after one hour of treatment. This phenomenon suggests that the loss of estrogenic activity of E2 and EE2 may result from the destructive cleavage of their aromatic ring structures. Therefore, future studies could focus on testing the hypothesis that the loss of estrogenic activity of the compounds is directly related to the breakage of the aromatic ring in their molecular structure. The literature (136) reports that Trametes versicolor can catalyze estrogen degradation using its produced laccase. However, the initial or direct metabolites involved in the degradation process are not explicitly described in this source. The final product of the complete degradation of E2 by *Steroidobacter denitrificans FS^T^ (* 137) is N_2_O. *Novosphingobium* sp. *ARI-1 (* 116, 138) has the ability to degrade E2 into compounds with significantly lower molecular mass or simple organic acids. *Pseudomonas aeruginosa TJ1 (* 139), *Rhodococcus zopfii Y50158*, along with *Rhodococcus equi Y50155, Y50156, and Y50157 (* 140), *Stenotrophomonas tumulicola ASc2, Serratia marcescens ASc5 (* 141), and *Rhodococcus equi DSSKP-R-001 (* 142), are capable of degrading E2. However, the specific pathways for E2 degradation in these microorganisms remain unclear, necessitating further in-depth studies and revelations.

### 3.2 Regulation of enzymatic activity in estradiol-degrading bacteria in natural environments

In natural environments, the enzyme activity of estradiol-degrading bacteria is regulated by various factors. An in-depth understanding of the specific regulatory mechanisms influencing the activity of these enzymes is crucial for adjusting the metabolic activities of gut microbiota to enable effective targeted interventions. **Table 2** summarizes the regulation of bacterial enzymatic activity involved in estradiol degradation within natural environments. Regulatory factors for estradiol-degrading enzymes include, but are not limited to, the following:

**Table 2:** Regulation of estradiol-degrading bacterial enzyme activity in natural environments.

Sample	Factor	Condition	Effect	Reference
Estradiol	Enzyme activity inhibitor	High concentration of isoflavones	Significantly inhibits 17β-HSD enzyme activity (Zn²^+^ inhibition rate: 62%), slowing E2 metabolism and degradation	(144)
Estradiol	Enzyme activity inhibitor	Zn²^+^, Fe²^+^, Fe³^+^, and other metal ions	Zn²^+^ shows the strongest inhibitory effect on E2 degradation enzyme 17β-HSD, with an inhibition rate of up to 62%	(146)
*Pseudomonas aeruginosa* TJ1 strain	Temperature	20-30°C	E2 degradation rate is the fastest within the 20-30°C range	(139, 148, 149)
*Microbacterium* sp. strain MZT7	Temperature	40°C	Enzyme activity is optimal between 20-40°C, with a degradation rate of up to 86.47%; activity significantly declines above 50°C and is completely lost at 70°C	(150)
Soil sample	Oxygen content	Aerobic and anaerobic environments	E2 half-life is 2.1 days in aerobic conditions, while it shortens to 1.6 days in some anaerobic samples, indicating higher degradation efficiency	(153)
Marine sediment	Oxygen content	Aerobic and anaerobic environments	E2 half-life is 4.4 days in aerobic conditions but extends significantly to 70 days in anaerobic environments	(154)
*Microbacterium resistens* MZT7	pH	pH 5-11	Enzyme activity is highest under alkaline conditions (pH 9), with a degradation efficiency of 86.55%; efficiency drops to 51.26% and 38.18% at pH 5 and 11, respectively	(150)
*Achromobacter xylosoxidans* and *Ralstonia* sp. *picketii*	Substrate concentration	Different initial E2 concentrations	E2 concentration variations have no significant effect on degradation rate	(146)
Activated sludge sample	Substrate concentration	Lower initial E2 concentration	Microbial degradation of E2 is more efficient under low concentration conditions	(163, 164)
*Bacillus* sp. E2Y4 strain	Substrate concentration	Different initial E2 concentrations	Degradation rate positively correlates with increased initial concentration	(129)

5-HT, 5-Hydroxytryptamine; GABA, γ-Aminobutyric acid; NE, Norepinephrine; DA, Dopamine; BDNF, Brain-Derived Neurotrophic Factor; E1, Estrone; DHT, Dihydrotestosterone; 4-OH-E1, 4-hydroxyestrone; 4-OH-E2, 4-hydroxyestradiol.

#### 3.2.1 Inhibitors of enzyme activity

Isoflavones are bioflavonoids abundant in soy and its derivatives, commonly consumed in daily diets (143). Keung (144) discovered that isoflavones can inhibit the γ-isozymes of ethanol dehydrogenase (ADH) *in vitro* in mammals. It is noteworthy that these γ-isozymes are responsible for catalyzing the oxidation process of 3β-hydroxysteroids. Subsequent studies have revealed that isoflavones not only exhibit inhibitory effects on γ-isozymes but also on β-hydroxysteroid dehydrogenase (β-HSD), thus slowing down the metabolic catabolism rate of steroid hormones (e.g., E2) by inhibiting 3β-HSD activity. Epidemiological evidence has indicated that high dietary intake of isoflavones correlates with a reduced prevalence of depressive symptoms (145). Therefore, dietary consumption of isoflavones may potentially alleviate depression by inhibiting the activity of estradiol-degrading enzymes in gut bacteria. Some researchers (146) discovered that Fe^2+^, Fe^3+^, Zn^2+^, and Cu^2+^ all significantly inhibit the activity of the estradiol-degrading enzyme 17β-HSD. Among these, Zn^2+^ exhibited the most potent inhibitory effect, reducing enzyme activity by 62%. Additionally, other studies (147) suggested that zinc deficiency might be a potential risk factor for depressive symptoms. Given this, a pertinent scientific question is whether specific molecular tools such as Zn2+ and isoflavones can be used as modulators of E2-degrading enzymes. Exploring their potential in depression interventions involves precise modulation of enzyme activity.

#### 3.2.2 Temperature

Among environmental factors, temperature directly influences the activity and stability of enzymes. Zeng et al. (139), in their investigation of the isolation of the *Pseudomonas aeruginosa TJ1* strain, proposed that the degradation of E2 occurred more rapidly within the temperature range of 20 to 30 degrees Celsius. This finding was corroborated by other researchers in their respective studies (148, 149), emphasizing the importance of optimal temperature as a key determinant for the efficient degradation of E2 by bacteria. It was reported (146) that the Microbacterium sp. strain MZT7, isolated from activated sludge in dairy farms, exhibited the highest activity of the estradiol-degrading enzyme 17β-HSD at 40°C. However, as the temperature surpassed 50°C, the enzyme activity displayed a notable decline, and it was entirely lost at 70°C. Another investigation (150) demonstrated that the degradation efficiency of the 17β-HSD enzyme from Microbacterium sp. MZT7 exceeded 50% within the temperature range of 20-40°C, reaching a peak degradation rate of 86.47%. In various segments of the intestine, local temperatures can fluctuate due to physiological functions and blood flow distribution. It has been observed (151) hat the stomach temperature is typically 0.2-0.6°C higher than that of the esophagus, which, in turn, is 0.3-1.0°C higher than the temperature in the axilla. Similarly, a canine study (152) yielded comparable findings: the luminal temperature in the duodenal region of dogs was approximately 6°C higher than the aortic temperature. Conversely, temperatures in the ileum, stomach, and large intestine regions, while relatively lower, remained around 0.5°C above the aortic temperature. This investigation underscores the presence of distinct temperature gradients within the mammalian digestive tract. Variances in gut temperature across different regions may impact the activities of estradiol-metabolizing enzymes within their resident microbial communities, suggesting a potential therapeutic avenue for addressing certain mental health disorders, such as depression in premenopausal women. However, the hypothetical connection and the precise pathways and mechanisms for clinical application require validation and further elucidation through rigorous scientific inquiry.

#### 3.2.3 Oxygen content

A study conducted by Deborah L. Carr (153)compared the biodegradation efficiency of 17β-estradiol in soil under aerobic and anaerobic conditions. It revealed that the half-life of 17β-estradiol was 2.1 days under aerobic conditions, whereas it decreased to 1.6 days under anaerobic conditions. This finding indicates that estradiol-degrading bacteria exhibit a higher degradation rate in anaerobic environments compared to aerobic ones in the soil samples examined. However, in a study conducted by Ying (154) on the biodegradation of E2 in marine sediments under both aerobic and anaerobic conditions, the findings contradicted those mentioned above. The experimental data revealed that the half-life of E2 degradation was 4.4 days under aerobic conditions, whereas it significantly extended to 70 days in anaerobic environments. This phenomenon was consistently corroborated in subsequent related studies by Ying (155). The reason for this difference may lie in the presence of enzymes that catalyze the oxidation reaction of E2 in aerobic bacteria, utilizing oxygen as the final electron acceptor. These enzymes are more active in aerobic environments. Conversely, anaerobic bacteria employ a different degradation mechanism, such as degradation through the reductive pathway or with the assistance of alternative electron acceptors like sulfate. Consequently, anaerobic bacteria demonstrate greater efficiency in degrading E2 under anoxic conditions. Notably, there are significant variations in the oxygen content of the intestinal lumen across different intestinal segments. One study (156) revealed that the partial pressure of oxygen in the distal small intestine (terminal ileum) of mice was extremely low, nearly zero (10 mm Hg), significantly lower than in the proximal digestive tract (60 mm Hg). This finding was corroborated by another study (157), which found that the partial pressure of oxygen in the mouse cecum was less than 1 mm Hg. The anaerobic environment in the distal intestinal lumen primarily arises from metabolic activities, including those of gastrointestinal (GI) microorganisms, oxidative reactions involving epithelial cells, and non-microbial sources such as lipid oxidation. Oxygen is continually depleted through these pathways, leading to a pronounced anaerobic environment in the region (156). Simultaneously, oxygen concentration serves as a pivotal factor influencing the structure of the gut microbial community (156, 157). Consequently, alterations in intestinal oxygen levels can affect both the composition of the gut microbial community and the activity of estradiol-degrading bacterial enzymes. Modulating oxygen levels in the gut could thus offer a prospective treatment avenue for certain mental health conditions, including depression in premenopausal women.

#### 3.2.4 PH

On one hand, enterobacteria residing in various segments of the intestinal tract may exhibit diverse E2 degradation activities due to variations in pH values. On the other hand, the intestinal microbiota can indirectly influence the functional activities of estradiol-degrading enzymes by actively regulating the acidity (pH) of their microenvironments (158). Estradiol-degrading enzymes from various microorganisms demonstrate varying activity levels across different pH environments. Hao et al. (146) observed that the estradiol-degrading enzyme 17β-HSD exhibited superior performance in alkaline conditions compared to acidic ones, with optimal catalytic activity occurring at pH 9. Additionally, the 17β-HSD enzyme derived from *Microbacterium resistens MZT7 (* 150) demonstrated the highest degradation efficiency of 86.55% at pH 7. However, at pH 5 and 11, the degradation efficiency of E2 dropped significantly to 51.26% and 38.18%, respectively. In the human gastrointestinal tract, various regions exhibit distinct pH values. The differences in pH across intestinal segments arise from a multitude of physiological and microbiological factors. These include the regulation of digestive fluid secretion and acid-base balance, the metabolic activities of intestinal microbiota, and the anatomical location with its associated physiological properties. In a study involving wasps (159), researchers found that *Lactobacillus Firm-5*, a member of the Lactobacillus genus, metabolizes lactic acid, leading to a reduction in the intestinal pH of wasps. Previous studies (160) have indicated that the human gastric pH is highly acidic, ranging from 1.0 to 2.5, while the pH of the proximal small intestine, terminal ileum, and cecum averages 6.6, 7.5, and 6.4, respectively. Moreover, there is a gradual increase in pH from the right colon to the left colon, averaging 7.0. Research (161) has demonstrated that pH not only influences microbial community structure but also modulates pH sensitivity through microbial and metabolic interactions among fermenting species. Hence, precise modulation of gut pH through dietary adjustments, probiotic supplementation, pharmacotherapy, and lifestyle optimization presents a promising therapeutic approach for managing mental health issues, including depression in premenopausal women. These interventions target micro-level modifications in the gut environment, potentially affecting mental health and mood regulation in the host. Future research should delve deeper into how these methods influence interactions between the gut microbiota and the host nervous system, and their viability in treating mental disorders.

#### 3.2.5 Substrate concentration

In terms of substrate concentration, while isolation studies of Achromobacter xylosoxidans and *Ralstonia* sp. *picketii (* 162) suggested that changes in E2 concentration did not significantly affect the rate of degradation, Ternes et al. (163, 164) found in 1999 that under lower concentration conditions, microorganisms in activated sludge degraded E2 more efficiently. Additionally, Jiang et al. (129) observed in their study that the *Bacillus* sp. *E2Y4* strain exhibited a positive correlation between the rate of E2 degradation and progressively higher initial steroid concentrations. The variation in the degradation rate of E2 by microorganisms in response to substrate concentration may be attributed to differences in enzyme properties, metabolic mechanisms, and ecological niche competition among different strains. In conclusion, varying substrate concentrations may regulate the enzyme activity of estradiol-degrading bacteria, thereby influencing their degradation rate performance.

In summary, the degradation rate of estradiol (E2) by microorganisms in nature depends significantly on the enzymatic activities within their bodies, which are influenced by various factors such as enzyme inhibitors, temperature, oxygen availability, pH, and substrate concentrations. By thoroughly investigating and modeling the biodegradation mechanisms and enzyme activities existing in nature, we may uncover innovative strategies to precisely regulate the enzymes involved in E2 metabolism within the gut microbial community. This approach aims to intervene in gut microecological processes that could potentially lower E2 levels associated with depression development, offering new perspectives on addressing this complex physiopathological condition.

## 4 The impact of microbial degradation products of estradiol on depression in natural environments

In nature, microbial metabolism of E2 primarily yields E1, alongside DHT, 4-OH-E1, 4-OH-E2, pyridinestrone acid, 2,3-SAOA, HIP, and other ring-cleavage products. Currently, potential associations between additional metabolites (e.g., pyridinestrone acid) beyond E1, DHT, 4-OH-E1, and 4-OH-E2 and depression remain unexplored. **Table 1** summarizes the mechanistic impact of estradiol metabolites on the pathogenesis of depression.

### 4.1 Estrone

E1, an endogenous estrogen, is synthesized in humans and animals through the metabolic conversion of E2. Unlike its precursor E2, E1 exhibits relatively low biological activity (165). It constitutes a significant portion of Premarin, a hormone replacement therapy (HRT) (166) drug widely employed to mitigate menopausal symptoms affecting both physical and cognitive functioning in peri- and postmenopausal women. Thomson et al. (167) noted in a controlled trial involving perimenopausal women that E1 positively impacted sleep quality within this demographic, indicated by a decrease in nocturnal awakenings. However, regarding the alleviation of psychological symptoms like depression and anxiety, the trial results did not demonstrate a significant difference in the efficacy of E1 compared to placebo. It has been demonstrated that E1 exhibits a neuroprotective effect under certain conditions (168, 169). However, a study comparing the impact of 17β-estradiol and E1 on newly generated neurons revealed that 17β-estradiol notably enhances the survival of these neurons and improves their response to spatial memory tasks (170). Conversely, E1 was found to significantly decrease the survival of newly generated neurons within the dentate gyrus region. Thus, the highly active form of E2 demonstrates protective effects against depression. However, when E2 undergoes microbial or metabolic processes in the body and is converted to the weaker or even harmful neuroprotective E1, it may adversely affect the human nervous system, potentially contributing to the development of depression.

### 4.2 Dihydrotestosterone

DHT is a naturally occurring androgen in the human body, resulting from the 5α-reductase-catalyzed conversion of testosterone (T) in target organs like the prostate, skin, and liver. It exhibits higher biological activity than T. Neuroinflammation has been implicated (171) as a central factor in the development and progression of various neurodegenerative diseases, including Alzheimer’s disease (AD), Parkinson’s disease (PD), and others. A study by Yang (172) demonstrated that DHT has protective effects in a mouse model of neuroinflammation induced by lipopolysaccharide (LPS). Subsequent investigations showed that DHT could enhance spatial learning and memory in LPS-treated mice while also restoring motor coordination and partially reversing impaired motor activity. These findings suggest that DHT possesses substantial anti-neuroinflammatory and neuroprotective properties. It has also been suggested (173) that serum or tissue levels of 5α-dihydrotestosterone (5α-DHT) may correlate with the severity of depressive symptoms in certain individuals. The question arises whether the potential ‘loss’ resulting from E2 metabolism can be offset by the biological ‘gain’ from newly generated DHT during their conversion. This inquiry necessitates rigorous experimental design and quantitative analysis to evaluate the metabolic balance during the conversion of E2 to DHT and its comprehensive impact on physiological functions.

### 4.3 4-hydroxyestrone (4-OH-E1)

4-OH-E1, a metabolite of the natural estrogen E1, demonstrates significant bioactivity in tissues such as the mammary gland and uterus. Within endocrinology, 4-OH-E1 serves as a crucial indicator of estrogen metabolic activity and its impact on health. In a study utilizing mouse hippocampal neuronal cells as an *in vitro* model (174), researchers evaluated 25 different endogenous estrogen metabolites and made a surprising discovery: despite its relatively weak estrogenic activity compared to E1, 4-OH-E1 displayed the most remarkable protective effect against oxidative stress-induced neurotoxicity. This protective effect surpassed even that of the highly estrogenic 17β-estradiol. Notably, this conclusion extends beyond *in vitro* models, as 4-OH-E1 also demonstrated superior protection to 17β-estradiol in rat hippocampal tissues subjected to kanamycin-induced oxidative damage. 4-OH-E1 plays a distinctive role in neuroprotective functions (174) by mediating the process of p53 deacetylation through the SIRT1 protein. This biochemical process leads to an enhanced spatial transfer of p53 protein from the nucleus to the cytoplasm, suggesting that 4-OH-E1 could positively influence the nervous system by regulating the subcellular localization and activity of the key transcription factor p53. Based on the above information, 4-OH-E1 may exert more significant positive effects on neurological function compared to E2 of the same molecular weight. This difference could partially explain why some non-menopausal women maintain good mental health without depressive symptoms despite lower E2 levels. However, this hypothesis requires validation through further scientific studies, including in-depth exploration of the mechanisms through which 4-OH-E1 and E2 contribute to neuroprotection and mood regulation.

### 4.4 4-hydroxyestradiol (4-OH-E2)

4-OH-E2 is an endogenous metabolite of E2, catalyzed by the cytochrome P450 enzyme system in the human body. It exhibits potent estrogenic activity and has a high binding affinity to the estrogen receptor (175, 176). In a study (177), 4-OH-E2 was found to induce cell proliferation in the subventricular zone (SVZ) brain region of female rats, but this stimulatory effect was not observed in pituitary cells. This suggests a tissue- or organ-selective mechanism of action for 4-OH-E2. In another neonatal mouse experiment (178), 4-OH-E2 was shown to up-regulate NE levels in the hypothalamus of neonatal female rats. Previous studies have suggested that E2 can exert antidepressant effects by increasing NE concentrations, thus prompting speculation that 4-OH-E2 may possess similar antidepressant properties. However, other research indicates that 4-OH-E2 exhibits chemical reactivity and may cause genotoxicity and mutagenicity (179, 180). In conclusion, further scientific validation is needed to determine whether 4-OH-E2 can serve as an antidepressant and elucidate its specific mechanism of action.

Taken together, it is reasonable to hypothesize that there may be an, as yet, undefined link between microbial-mediated degradation products of E2 in nature and depression. This hypothesis provides a crucial theoretical framework and research direction for exploring how gut microbes influence the development of depression by modulating E2 metabolism. It also underscores the need for caution when evaluating the effects of E2 degradation on depression, as the dynamic balance between decreasing E2 levels and accumulating degradation products could result in diverse biological effects.

## 5 The effect of gut microorganisms on estradiol

The human body harbors a vast array of microorganisms, with nearly 90% of them (approximately 10 to 100 trillion) residing in the gastrointestinal tract (181). Within the gastrointestinal tract, there are over 500 different species of bacteria, comprising approximately 1 kilogram of the body’s weight, and the human intestine harbors approximately 10^11^ bacteria per gram (182–185). Bacteroidetes and Firmicutes typically dominate, with Proteobacteria, Verrucomicrobia, Actinobacteria, Clostridia, and Cyanobacteria following suit (186–188). The intestinal microbiota exerts regulatory effects on steroid hormones like E2, which can indirectly or directly influence the *in vivo* concentration of E2.

### 5.1 The gut microbiota regulates mechanisms of estradiol metabolism through synthetic enzymes

**Figure 3:**
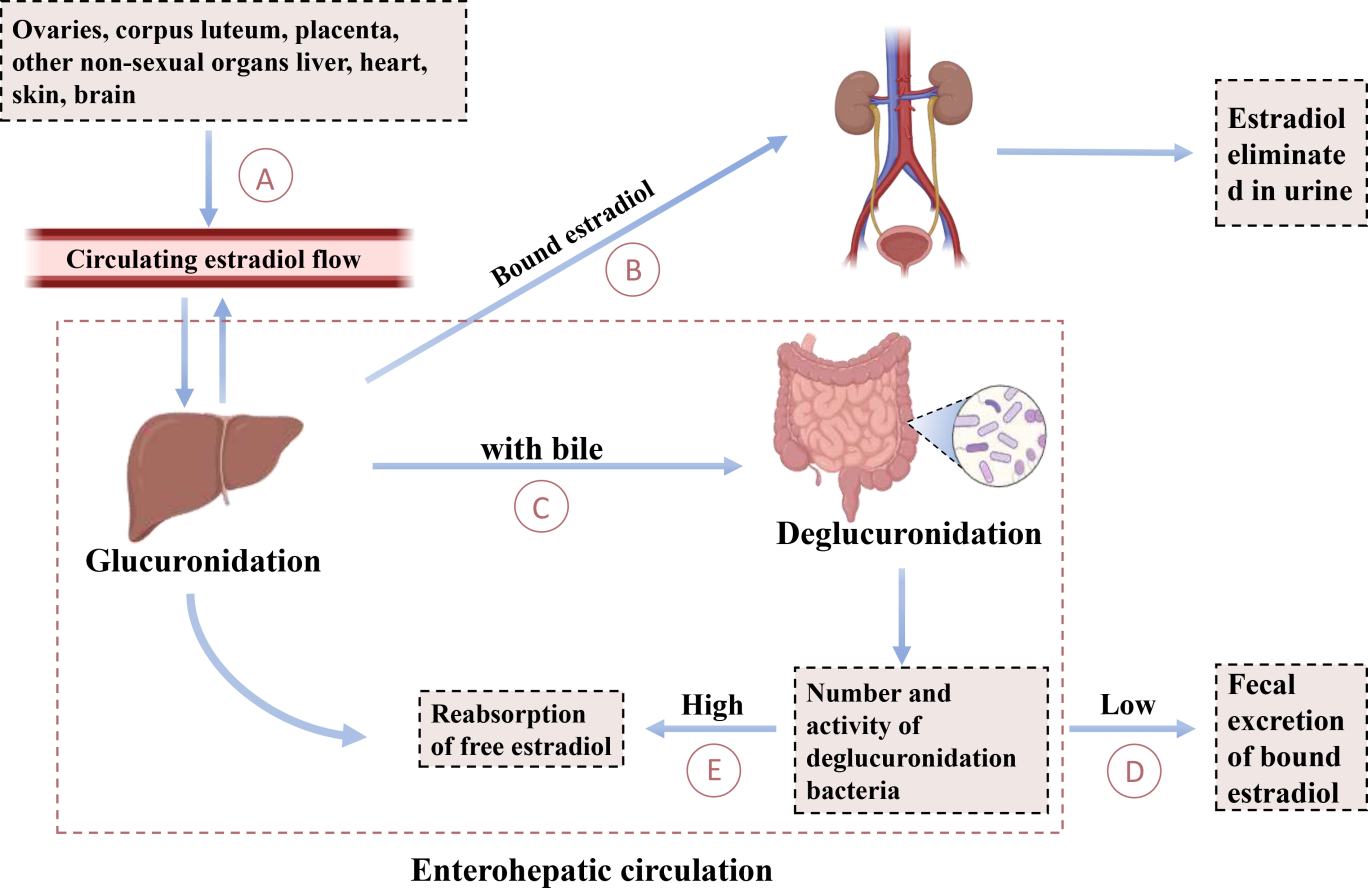
Enterohepatic circulation of estradiol. This diagram explains the cycling of estradiol between the gut and the liver. **(A)** The production of estradiol by the ovaries, corpus luteum, placenta, and other nonsexual organs such as the liver, heart, skin, and brain regulates circulating estradiol flow. **(B)** A portion of bound estradiol is excreted as waste through the kidneys. **(C)** A portion of bound estradiol is excreted through the biliary tract into the intestine. **(D)** Deglucuronidated bacteria are excreted via fecal excretion when their numbers and activity are low. **(E)** Free estradiol is reabsorbed into the liver when the number and activity of de-glucuronidating bacteria are high.

Recent studies have shown that gut microbes can modify the activity of steroid hormones through metabolism, thereby affecting serum steroid levels (189). This indicates that gut microbes not only influence the gut’s internal environment but also potentially impact systemic physiological functions via hormonal signaling pathways, including hormones associated with mood and mental health, such as E2. In the body, E2 undergoes hepatic metabolism, wherein it combines with sulfate and glucuronic acid to form water-soluble compounds. These conjugates are primarily excreted as waste products via the kidneys, although a portion is also eliminated through the biliary tract into the intestines (190). Enzymes produced by certain intestinal microbiota further modify the serum steroid hormone levels by altering the ratio of active to inactive compounds (191), a point supported by other researchers (192). This process, illustrated in **Figure 3**, is known as the liver-gut cycle, suggesting the involvement of intestinal microbiota’s glucuronidase activity in estrogen metabolism. Specific gut microbiota species possess enzyme systems capable of metabolizing and converting steroid hormones, including E2. For instance, the intestinal microorganism Eggerthella lenta strain C592 contains 17β-hydroxysteroid dehydrogenases (17β-HSD), which catalyzes the oxidative conversion of biologically active E2 to less active E1 (193). Recent studies have revealed that the gut microbiota, specifically Klebsiella aerogenes, degrades E2 through the expression of 3β-hydroxysteroid dehydrogenases (3β-HSD) (39). Conversely, β-Glucuronidase (gmGUS), present in Clostridium perfringens, has the capability to reactivate estrone-3-glucuronide and estradiol-17-glucuronide into E1 and E2 (194). This process increases the proportion of E2 activity in the gut, facilitating the reabsorption of E2 into the bloodstream.

### 5.2 The intestinal microbiota influences the function of the hypothalamic-pituitary axis, thereby impacting estradiol levels

In the hypothalamic-pituitary-gonadal (HPG) axis, which regulates reproductive hormone levels and cyclic physiological processes, the hypothalamus secretes gonadotropin-releasing hormone (GnRH) to stimulate the anterior pituitary to release follicle-stimulating hormone (FSH) and luteinizing hormone (LH). FSH promotes the growth and development of ovarian follicles and stimulates granulosa cells to secrete estrogen, such as estradiol (E2); meanwhile, LH facilitates corpus luteum formation during follicular maturation, further enhancing E2 secretion. Recent studies have shown that endotoxins, such as lipopolysaccharides (LPS), can inhibit GnRH secretion and reduce LH secretion by the pituitary, leading to a significant decrease in E2 levels (195–197). Additionally, germ-free mice exhibit significant impairments in working memory, and antibiotic-treated mice show a notable decline in object recognition ability (198, 199). These findings suggest that gut microbiota may regulate E2 levels through the HPG axis, although the specific molecular mechanisms remain unclear.

Further studies revealed that gut microbiota-derived short-chain fatty acids (SCFAs), such as acetate, propionate, and butyrate, can regulate hypothalamic-pituitary-adrenal (HPA) axis activity by reducing GnRH secretion, thereby inhibiting LH and FSH secretion, delaying gonadal development, and decreasing estradiol levels (200). Furthermore, gut microbial metabolites (e.g., SCFAs and bile acids) and inflammatory signals play important roles in the regulation of HPA axis activity (201, 202). Dysbiosis of the gut microbiota may trigger chronic inflammation, activating the HPA axis, resulting in abnormal cortisol secretion, which further suppresses estradiol production.

In summary, gut microbiota regulate estradiol levels by influencing the activity of the HPA and HPG axes through metabolites (e.g., SCFAs) and inflammatory signals. This regulatory mechanism may provide potential therapeutic targets for diseases such as depression. However, further research is required to elucidate the specific molecular mechanisms and assess their clinical application value.

## 6 Gut microbiota affects depression through estradiol metabolism

Metchnikoff proposed a theory over 100 years ago suggesting that abnormalities in gut microbiota could underlie depression, anxiety, and other mental health issues. He advocated for addressing these disorders through probiotic supplementation. However, due to the limitations in science, technology, and research methods of that era, this theory was not thoroughly investigated or emphasized (203). Advances in science and technology, particularly in microbiome research, have prompted a reevaluation and validation of Metchnikoff’s theory. Since 2009, scientists have focused on investigating the role of the brain-gut axis in depression (204). Recent clinical studies have highlighted differences in gut microbiota between depressed patients and healthy individuals (205). Research indicates that depressed patients exhibit decreased α-diversity and β-diversity in their intestinal microbiota compared to controls (206, 207). Moreover, further analysis identified elevated levels of Actinobacteria and Fusobacteria at the phylum level (208). Additionally, multiple studies have observed reduced E2 levels in depressed premenopausal women (21). Studies using rodent models have convincingly shown that E2 plays a crucial role in maintaining emotional balance and psychological well-being; decreased serum E2 levels in experimental animals lead to depressive-like behaviors (209). Furthermore, recent research (189) has uncovered the ability of gut microbes to influence steroid hormone activity through synthetic or metabolic pathways, indicating a novel role in hormone regulation beyond the gut environment. This suggests that gut microbes not only impact gut health but also influence broader physiological functions via hormone signaling pathways. These findings shed light on the connection between gut microbes and various health conditions, including psychiatric disorders like depression. Despite advancements, the precise mechanisms by which gut microbiota metabolize E2 and contribute to depression remain unclear. In recent years, Di Li (39) and colleagues conducted a study on women with perimenopausal depression and identified a specific bacterium, Clostridium perfringens, in the gut microbiota of these patients. Furthermore, the researchers isolated a specific estradiol-degrading enzyme, 3β-HSD, from this bacterium (39). The study successfully transfected the 3β-HSD gene into Escherichia coli. Subsequently, these transgenic bacteria were used to administer treatment to de-ovulated rats via gavage. Experimental results demonstrated a notable reduction in serum and brain tissue E2 concentrations in treated rats, concomitant with the emergence of depression-like behavioral traits. These observations suggest that Clostridium perfringens might have the capability to degrade E2 through the expression of 3β-HSD, potentially contributing to depression development. Furthermore, it is essential to investigate the presence of other estradiol-degrading bacteria akin to Clostridium perfringens in the intestinal microecosystem, as well as explore alternative estradiol-degrading enzymes resembling 3β-HSD. Understanding their impact on depression represents a crucial avenue for future research.

## 7 Conclusions

This paper systematically reviews the molecular mechanisms of E2 action in the pathophysiology of depression and provides insights into recent research advances concerning the regulation of E2 metabolism by gut microbial communities. Additionally, it synthesizes and analyzes the mechanisms of E2 degradation by various microorganisms in natural environments and evaluates the scientific evidence available for a potential link between these metabolites and depression. Despite the progress in existing studies, several critical questions remain that require thorough exploration in future research endeavors.

1. In recent years, preliminary results have emerged in exploring the role of gut microbiota in E2 metabolism and its implications in depression pathogenesis. However, extensive research remains to fully elucidate this area. Drawing from case studies on microbial degradation of E2 and its products in natural environments may offer crucial insights into how gut microbes modulate E2 levels via estradiol-degrading enzymes, thereby influencing the onset and progression of depression.
2. Numerous scientific studies have investigated the degradation of E2 by microorganisms in natural environments, offering valuable insights and a robust theoretical framework for understanding the intricate process of E2 metabolism by intestinal microbiota. Nonetheless, a significant research gap persists in this area concerning the identification of initial degradation products and key enzymes. Consequently, further screening and identification efforts are imperative to delve deeper into the degradation mechanism of microbial strains, elucidate the detailed metabolic pathways, and elucidate the functions of pivotal enzymes involved.
3. Gut bacteria can degrade E2, leading to three scenarios of degradation product formation. First, metabolites such as E1 and 4-OH-E2 may arise, which are less neuroprotective or potentially harmful compared to E2 itself, thus negatively impacting depression. Second, the production of metabolites like 4-OH-E1, which are more neuroprotective than E2, might explain why some premenopausal women with reduced estradiol levels do not experience depression symptoms, although further research is needed to validate this hypothesis. Third, metabolites such as DHT may possess similar or unspecified neuroprotective effects as E2. However, a “balance” issue emerges: for every molecule of E2 degraded, a corresponding molecule of degradation products is generated. Whether the benefit from these products compensates for the loss of E2’s protective effect on depression warrants investigation. Understanding how gut microbial transformation products influence body functions through specific mechanisms and their role in both normal physiology and disease pathology constitutes a crucial area for future research.
4. Gut microbiota capable of degrading estradiol via 3β-HSD may exist in women with depression, potentially contributing to the development of the condition. However, whether similar gut microbiota and their degrading enzymes exist in men with depression, influencing the disease progression through the endocrine pathway, remains an under-explored area that requires further investigation.

## Glossary

AD: Alzheimer’s disease
AMO: Ammonia monooxygenase
BDNF: Brain-Derived Neurotrophic Factor
BNST: Bed nucleus of the stria terminalis
CNS: Central nervous system
CREB: Cyclic AMP Response Element Binding Protein
DA: Dopamine
DAT: Dopamine Transporter
DHT: Dihydrotestosterone
ERE: Estrogen Response Element
E0: Estratetraenol
E1: Estrone
E2: Estradiol
FSH: Follicle-Stimulating Hormone
GABA: γ-aminobutyric
GM: Gut Microbiota
nRH: Gonadotropin-releasing hormone
GPCRs: G-protein-coupled receptors
GuGUS: β-Glucuronidase
HPA: Hypothalamic-pituitary-adrenal
HPLC: High-performance liquid chromatography
HRT: Hormone replacement therapy
Kiss1: Kisspeptin
LH: luteinizing hormone
LPS: lipopolysaccharide
IGF-1: Insulin-like Growth Factor 1
IP3: Inositol 1,4,5-Trisphosphate
IP3R: Inositol 1,4,5-Trisphosphate Receptor
MAOIs: Monoamine Oxidase Inhibitors
MAPK: Mitogen-activated protein kinase
NE: Norepinephrine
NET: Norepinephrine Transporter
PD: Parkinson’s disease
POA: Preoptic area
SVZ: Subventricular zone
T: Testosterone
TCAs: Tricyclic Antidepressants
TCA: cycle Tricarboxylic acid cycle
WHO: The World Health Organization
3β-HSD: 3β-hydroxysteroid dehydrogenase
4-OH-E1: 4-hydroxyestrone
4-OH-E2: 4-hydroxyestradiol
5α-DHT: 5α-dihydrotestosterone
5-HT: 5-hydroxytryptamine
17β-HSD: 17β-hydroxysteroid dehydrogenase
β-HSD: β-hydroxysteroid dehydrogenase
