# Supplementary material for: Estradiol metabolism by gut microbiota in women’s depression pathogenesis: inspiration from nature
Source: Front Psychiatry. 2025 Jan 28;16:1505991. doi: 10.3389/fpsyt.2025.1505991 (PMC11811108; doi:10.3389/fpsyt.2025.1505991)
Supplement: Supplementary file 1 [file Table1.docx]

| **Strains** | **Initial product** | **Enzyme** | **Other metabolic products** | **Bibliography** |
| --- | --- | --- | --- | --- |
| Sphingomonas sp. KC8 | E1 | 17β-hydroxysteroid dehydrogenase、 flavin-dependent monooxygenase (estrone 4-hydroxylase)、 extradiol dioxygenase (4-hydroxyestrone  4,5-dioxygenase) | 4-hydroxyestrone、ring-cleavage product、pyridinestrone acid | ^[122-124]^ |
| Acinetobacter sp.DSSKY-A-001 | E1 | catechol 1,2-dioxygenase、dioxygenase、7-α-hydroxysteroid dehydrogenase | R2、R3 | ^[125]^ |
| Rhodococcus sp. DS201 | E1 |  | 4-OH-E1、M1、M2、5-(4-(2-carboxyethyl)-7a-methyl-1-oxooctahydro-1H-inden-5-yl)pent-4-enoic acid、pent-4-enoic acid、3-(7a-methyl-1,5-dioxooctahydro-1H-inden-4-yl) propanoic acid、5-hydroxy-4-(3-hydroxypropyl)-7a-methyloctahydro-1H-inden-1-one、2-ethyl-3-hydroxy-6-methylcyclohexane-1-carboxylic acid | ^[126]^ |
| Sphingomonas sp. ED8 | E1 | Oxidase | 4-OH-E1 | ^[118]^ |
|  | 4-OH-E2 |  |  | ^[118]^ |
|  | hydroxy-E2 |  | keto-E2、7-keto-E1、3-(4-hydroxyphenyl) -2-hydroxyprop-2-enoic acid | ^[118]^ |
| Aminobacter  strains KC6、KC7 | E1 | Nonspecific monooxygenase |  | ^[124]^ |
| Sphingomonas sp. CYH | E1 |  |  | ^[119]^ |
| Ralstonia pickettii BP2 | E1 |  |  | ^[117]^ |
| Phyllobacterium | E1 |  |  | ^[117]^ |
| Flavobacterium strain KC1 | E1 |  |  | ^[124]^ |
| Nocardioides strain KC3 | E1 |  |  | ^[124]^ |
| Rhodococcus strain KC4 | E1 |  |  | ^[124]^ |
| Microbacterium strain KC5 | E1 |  |  | ^[124]^ |
| Sphingomonas strains KC9、KC10、  KC11、KC14 | E1 |  |  | ^[124]^ |
| Brevundimonas strain KC12 | E1 |  |  | ^[124]^ |
| Escherichia strain KC13 | E1 |  |  | ^[124]^ |
| Brevundimonas diminuta I | E1 | 17β-hydroxysteroid dehydrogenase |  | ^[127]^ |
| Virgibacillus halotolerans LF1 | E1 |  |  | ^[128]^ |
| Bacillus flexus LF3 | E1 |  |  | ^[128]^ |
| Bacillus licheniformis LF5 | E1 |  |  | ^[128]^ |
| Bacillus sp. E2Y1、E2Y2、E2Y3、E2Y4、E2Y5 | E1 |  |  | ^[129]^ |
| Ammonia-oxidizing bacterium Nitrosomonas  europaea | E0 | Ammonia monooxygenase |  | ^[131,132]^ |
| Denitratisoma  sp. strain DHT3 | Dihydrotestosterone | Cobalamin-dependent methyltransferase | 17β-hydroxy-1-oxo-2,3-seco-androstan-3-oic acid (2,3-SAOA) 、 3aα-H-4α(3′-propanoate)-7aβ-methylhexahydro-1,5-indanedione (HIP) | ^[133]^ |
| Denitratisoma oestradiolicum AcBE2-1^T^ |  | Cytochrome  oxidase | CO_2_、H_2_O | ^[134]^ |
| Ammonia-oxidizing bacterium White rot fungi |  | Ligninolytic  enzymes、 laccase |  | ^[135]^ |
| Trametes versicolor |  | Laccase |  | ^[136]^ |
| Asteroidobacter denitrificans FS^T^ |  |  | N_2_O | ^[137]^ |
| Novosphingobium sp. ARI-1 |  |  | Low molecular mass compounds（CO_2_）、simple organic acids | ^[116,138]^ |
| Pseudomonas aeruginosa TJ1 |  |  |  | ^[139]^ |
| Rhodococcus zopfii Y50158 |  |  |  | ^[140]^ |
| Rhodococcus equi Y50155、Y50156、Y50157 |  |  |  | ^[140]^ |
| Stenotrophomonas tumulicola ASc2 |  |  |  | ^[141]^ |
| Serratia marcescens ASc5 |  |  |  | ^[141]^ |
| Rhodococcus equi DSSKP-R-001 |  |  |  | ^[142]^ |

**Supplementary Table 1**. Microorganisms involved in estradiol degradation and their metabolites, primary products, and enzymes associated with primary products.

Estrone(E1) ,Ammonia monooxygenase (AMO) ,Dihydrotestosterone（DHT）,4-hydroxyestrone (4-OH-E1),4-hydroxyestradiol (4-OH-E2), 1,3 ,5(10),16-tetraen-3-ol (estratetraenol,E0
